# C-Reactive Protein-to-Albumin Ratio and Hospital-Recorded Mortality in Patients with ICD-10-Coded Acute Kidney Injury: A Retrospective Cohort Study

**DOI:** 10.3390/jcm15218422

**Published:** 2026-10-29

**Authors:** Sema Ayten, Vehbi Özaydın, Hülya Yılmaz Başer, Maciej Maslyk, Lukasz Szarpak, Onur İncealtin, Cihad Ünsal Karahaliloğlu, Michal Pruc

**Affiliations:** 1 Department of Emergency Medicine, İstanbul Göztepe Prof. Dr. Süleyman Yalçın City Hospital, 34722 Istanbul, Türkiye; 2 Department of Emergency Medicine, Bandırma Onyedi Eylül University, 10200 Bandırma, Türkiye; ylmz_hly_35@yahoo.com; 3 Institute of Biological Sciences, The John Paul II Catholic University of Lublin, 20-708 Lublin, Poland; 4 Institute of Medical Science, Collegium Medicum, The John Paul II Catholic University of Lublin, 20-708 Lublin, Polandmichal.pruc@kul.pl (M.P.); 5 Henry J.N. Taub Department of Emergency Medicine, Baylor College of Medicine, Houston, TX 77030, USA

**Keywords:** acute kidney injury, albumin, C-reactive protein, C-reactive protein-to-albumin ratio, mortality, retrospective cohort

## Abstract

**Background**: The prognostic value of the C-reactive protein-to-albumin ratio (CAR) relative to its components in acute kidney injury (AKI) is uncertain. **Methods**: We analyzed 7181 patient-date encounters (5744 patients; 2016–2025) identified using ICD-10 codes N17.x after exclusion of coded chronic kidney disease; AKI could not be verified against KDIGO criteria. The primary outcome was hospital-recorded death within 30 days, with 180-day mortality exploratory. Age- and sex-adjusted associations and discrimination of CAR, C-reactive protein (CRP), and albumin were evaluated in a common complete-marker sample, with sensitivity and patient-level bootstrap analyses. **Results**: CAR was available in 6603 encounters; hospital-recorded 30-day mortality was 9.6%. Each doubling of CAR was associated with higher odds of recorded death (adjusted odds ratio 1.25, 95% confidence interval 1.20–1.29). Areas under the receiver operating characteristic curve were 0.722 for albumin, 0.659 for CAR, and 0.642 for CRP. Separately modeled components outperformed CAR in adjusted models (0.737 versus 0.688; bootstrap 95% confidence interval for the difference, 0.034–0.065). CAR performance was attenuated in hospitalized-only analyses. **Conclusions**: CAR was associated with hospital-recorded mortality in code-identified AKI but did not outperform albumin. External validation is required; out-of-system deaths may have been missed. CAR should not be used as a stand-alone clinical decision tool.

## 1. Introduction

Acute kidney injury (AKI) is a heterogeneous clinical syndrome defined by an acute deterioration in kidney function, conventionally identified by changes in serum creatinine and/or urine output according to Kidney Disease: Improving Global Outcomes (KDIGO) criteria [1]. AKI ranges from transient functional impairment to severe structural kidney injury and can arise through hemodynamic, inflammatory, nephrotoxic, obstructive, ischemic, or mixed mechanisms. In the multinational Acute Kidney Injury–Epidemiologic Prospective Investigation (AKI-EPI) study, AKI occurred in more than half of critically ill patients and mortality increased across greater AKI severity [2]. Contemporary reviews further emphasize that AKI is not a single disease entity but a syndrome embedded in diverse clinical contexts, with prognosis shaped by the precipitating insult, baseline susceptibility, extra-renal organ dysfunction, and the trajectory of kidney recovery [3,4]. This biological and clinical heterogeneity helps explain why a single laboratory value rarely captures the full risk profile of an individual patient and provides a rationale for evaluating complementary prognostic biomarkers.

Systemic inflammation is relevant to many forms of AKI through endothelial and microcirculatory dysfunction, altered vascular permeability, immune-cell activation, oxidative stress, and direct or indirect tubular injury. C-reactive protein (CRP) is a widely available acute-phase reactant that rises in response to infection, tissue injury, ischemia, and other inflammatory states. Its accessibility makes it attractive for risk stratification, but CRP is highly nonspecific and its prognostic performance varies across clinical settings. A systematic review and meta-analysis of critically ill patients with sepsis found that baseline CRP was not consistently associated with mortality after accounting for important covariates [5], while the prospective French and European Outcome Registry in Intensive Care Units (FROG-ICU) study found that interleukin-6 provided stronger prognostic information than CRP for 90-day survival and organ dysfunction [6]. These observations support interpreting CRP as one component of an overall illness phenotype rather than as an isolated mortality marker.

Albumin may provide complementary information. It is the most abundant circulating plasma protein and a negative acute-phase reactant, and its concentration is influenced by inflammation, capillary permeability and redistribution, hepatic synthesis, renal or gastrointestinal losses, extracellular volume, and chronic disease. Importantly, serum albumin should not be interpreted as a direct biochemical measure of protein-energy malnutrition; the American Society for Parenteral and Enteral Nutrition emphasizes that low albumin primarily reflects inflammation and illness-related risk rather than nutritional status itself [7]. Mechanistic work similarly describes hypoalbuminemia as a consequence of inflammatory changes in vascular permeability, distribution volume, and albumin turnover rather than simply reduced protein intake [8]. In AKI, hypoalbuminemia has nevertheless shown consistent prognostic relevance: a meta-analysis of observational studies found low albumin to be associated both with the development of AKI and with mortality among patients with established AKI [9]. Thus, albumin may integrate several dimensions of acute and chronic vulnerability that are only partly captured by inflammatory markers such as CRP.

Because CRP and albumin reflect overlapping but biologically distinct aspects of acute illness, several groups have evaluated the CRP/albumin ratio (CAR) as a combined prognostic marker. In critically ill patients, CAR has been associated with mortality and has sometimes outperformed CRP alone [10]; CAR measured at intensive care unit (ICU) discharge has also predicted 90-day mortality in sepsis [11]. In AKI specifically, higher CAR has been associated with mortality in critically ill cohorts [12], and a multicenter study of 3995 patients with severe AKI requiring continuous renal replacement therapy found that CAR was associated with in-hospital mortality and added modest prognostic information to Acute Physiology and Chronic Health Evaluation II (APACHE II) or Sequential Organ Failure Assessment (SOFA) scores [13]. Associations have also been reported in broader AKI cohorts [14] and in severe sepsis or septic shock [15]. In hospital-acquired AKI, the related CRP/prealbumin ratio remained associated with mortality after adjustment for illness severity, whereas CAR itself did not retain the same independent association [16]. CAR has additionally been investigated as a predictor of AKI development after acute pancreatitis [17] and as a mortality marker in older emergency-department and hospitalized populations [18,19]. Taken together, these studies support a prognostic signal, but they do not establish that combining CRP and albumin into a ratio necessarily preserves or improves the information contained in the two components.

That distinction is important because ratio variables impose statistical as well as biological assumptions. On a logarithmic scale, log(CRP/albumin) is mathematically equivalent to log(CRP) minus log(albumin); a model using only log(CAR) therefore constrains proportional increases in CRP and decreases in albumin to enter with equal-magnitude, opposite-direction coefficients. More generally, ratio methods can obscure nonlinear relationships, amplify denominator-related variability, and create interpretive difficulties when the numerator and denominator contribute unequally to risk [20,21]. Recent epidemiologic discussion has likewise emphasized that ratios can be convenient for prognostic or predictive purposes while still combining component information under structural assumptions that can limit component-specific interpretation [21]. More recent simulation work, developed primarily for causal regression settings, has further illustrated that ratio terms do not substitute for the information contained in their component variables and that omission of component main effects can distort inference in those settings [22]. Although those results are not directly transferable to a prognostic comparison such as the present study, they reinforce the value of evaluating the underlying components alongside the ratio rather than assuming that a ratio is intrinsically superior. Direct, same-sample comparison of CAR with CRP and albumin is therefore necessary before concluding that the composite ratio is preferable. Most AKI-specific CAR studies have focused on intensive care or other highly selected populations, and such comparisons remain limited in broader hospital cohorts. We therefore examined the association of CRP, albumin, and CAR with hospital-recorded 30-day mortality in adults with an N17-coded diagnosis of AKI after source-level exclusion of records with documented N18.x CKD. The principal comparative question was whether CAR outperformed albumin or CRP in the same observations and whether adding CAR to a basic age- and sex-adjusted model improved apparent discrimination more than adding the individual components. Hospital-recorded 180-day mortality was retained as a supportive exploratory outcome. Importantly, AKI was an eligibility-defining diagnosis, not an outcome modeled in this study; we did not assess incident AKI or any causal effect of CAR on kidney injury.

## 2. Materials and Methods

### 2.1. Study Design and Setting

This retrospective, exploratory cohort study used routinely collected hospital data. Adult patient-date encounters involving individuals aged 18 years or older who presented to the emergency department or were otherwise evaluated at Istanbul Göztepe Prof. Dr. Süleyman Yalçın City Hospital between 2016 and 2025 were identified in the electronic health record using International Classification of Diseases, Tenth Revision (ICD-10) codes N17.x (acute kidney failure): N17.9 (unspecified) in 5840 encounters (81.3%), N17.8 (other acute kidney failure) in 1289 (18.0%), N17.0 (with tubular necrosis) in 51 (0.7%), and N17.2 (with medullary necrosis) in 1. AKI identification was therefore administrative. The available dataset did not contain sufficient baseline creatinine or urine-output data to independently confirm KDIGO diagnostic criteria or AKI stage. Validation studies indicate that ICD-10-coded AKI can identify clinically recognized AKI with relatively high positive predictive value but has limited sensitivity for laboratory-defined AKI; accordingly, the present cohort should be interpreted as a code-identified AKI population rather than as all patients meeting biochemical AKI criteria [23,24]. Accordingly, ICD-10 codes N17.x were used to define the eligible cohort, whereas hospital-recorded mortality was the outcome evaluated in the association and discrimination analyses.

Encounters were included regardless of whether the patient was subsequently hospitalized or managed and discharged directly from the emergency department. The study was approved by the local Institutional Ethics Committee (Approval No. 2025/0130; 28 August 2025) and conducted in accordance with the Declaration of Helsinki. Given the retrospective design and use of de-identified data, the ethics committee waived individual informed consent. Reporting was guided by the Strengthening the Reporting of Observational Studies in Epidemiology (STROBE) statement and the Reporting of studies Conducted using the Observational Routinely-collected health Data (RECORD) extension [25,26].

### 2.2. Study Population

**Figure 1:**
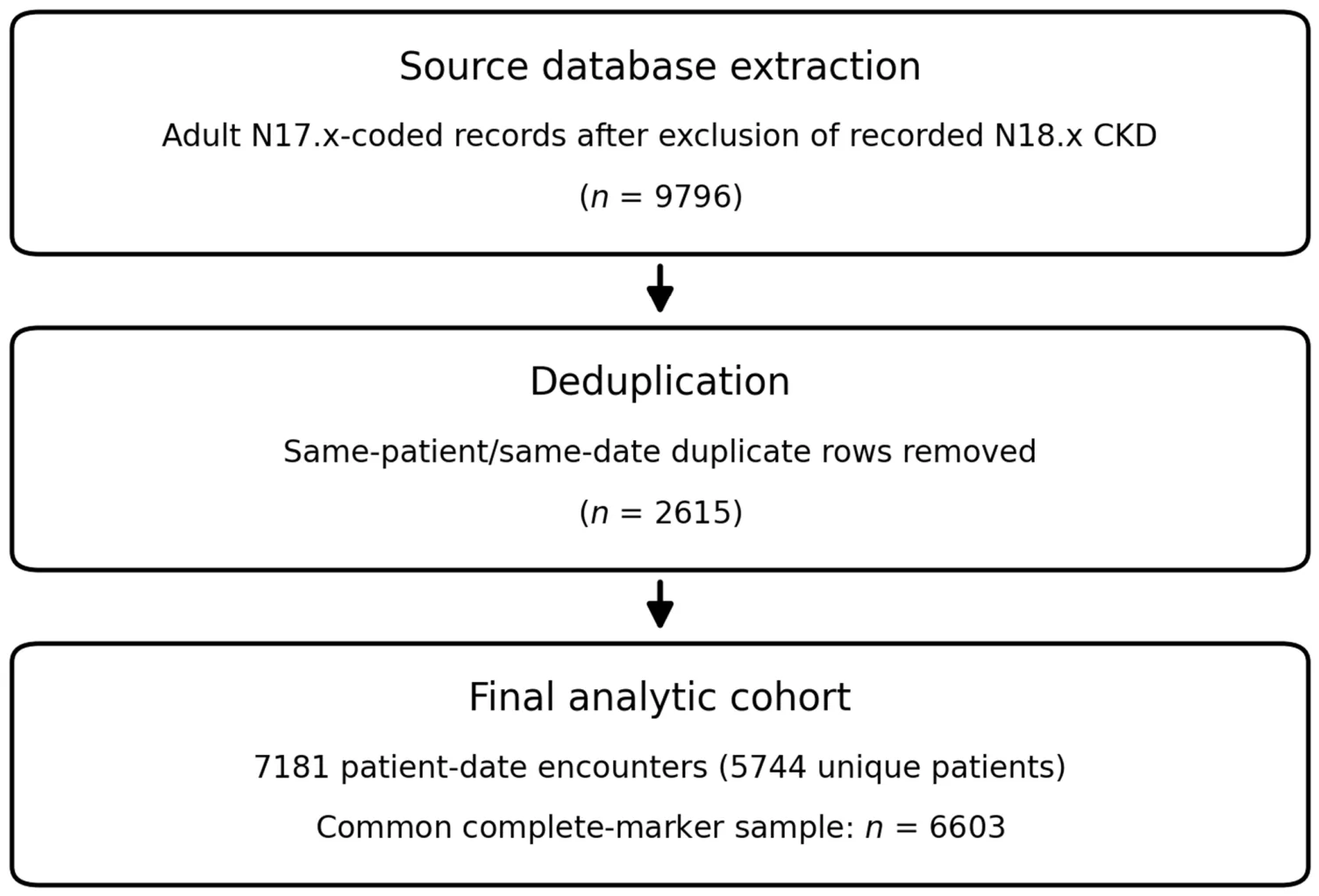
Study flow from source-database extraction to the final analytic cohort and common complete-marker sample. CKD, chronic kidney disease; CAR, C-reactive protein-to-albumin ratio.

At the initial data-extraction stage, records with a documented ICD-10 N18.x chronic kidney disease code were excluded from the source database query; accordingly, the extracted dataset of 9796 records had already been created after this administrative CKD exclusion. Duplicate applications were then defined using the combination of hospital patient number and application date. Same-patient/same-date applications were treated as duplicate representations of the same patient-date encounter; duplicate rows were removed so that one patient-date record remained, resulting in removal of 2615 rows. The duplicate groups were characterized against the raw source extract, and the record-selection rule is specified below. The final cleaning procedure deliberately did not use masked national identity numbers as the deduplication key because validation identified collisions between distinct patients after masking. Applications for the same patient on different dates were retained as separate eligible encounters, without assuming that every encounter represented a biologically independent AKI episode. The patient-date encounter was an operational unit defined by the combination of the hospital patient identifier and application date, rather than a clinically adjudicated admission or AKI episode. The available analytic documentation did not permit reliable classification of all repeated patient-date records as new hospital admissions, follow-up visits, or repeated assessments occurring during the same hospitalization. Therefore, observations from different dates were retained without assuming that they represented distinct admissions or independent AKI episodes. After deduplication, 7181 patient-date encounters representing 5744 unique patients remained; 1048 patients contributed more than one eligible encounter (up to 10 encounters per patient). The primary analytic unit was therefore the encounter. The CKD exclusion was based on recorded diagnostic coding at source extraction rather than systematic longitudinal estimated glomerular filtration rate (eGFR) or albuminuria ascertainment; consequently, unrecognized or uncoded chronic kidney disease and maintenance dialysis could not be fully excluded. The cohort derivation is summarized in Figure 1.

To ensure a reproducible deduplication procedure, the record-selection step was reconstructed from the raw source extract (9796 rows) and is fully specified here. Same-patient/same-date records formed 2031 duplicate groups comprising 4646 rows (maximum, six records per group). Within every duplicate group, the recorded values of albumin, CRP, urea, creatinine, age at diagnosis, sex, and death date were identical; the rows differed only in administrative application numbers and, in 23 groups, in hospital-admission linkage, where one row was linked to a hospital admission and the remaining row(s) were labelled as unmatched admissions. Diagnostic code and diagnosis text also differed among N17 subcodes in 338 groups, without affecting eligibility. Because the original deduplication rule had not been documented, a deterministic rule was specified retrospectively: within each patient-date group, the first record in source-file order was retained. Applying this rule to the raw extract, followed by the cleaning steps described in Section 2.3, regenerated the analytic dataset of 7181 encounters with complete agreement in all analysis variables (laboratory values, age, sex, admission status, and death dates) and reproduced the previously reported primary estimates. As a sensitivity check, alternative rules retaining the record with the lowest or the highest application number yielded identical laboratory, demographic, and mortality data; they differed only in the hospital-admission linkage of at most 18 encounters. This audit was performed independently in Python (version 3.12.3; Python Software Foundation, Wilmington, DE, USA) and in R (version 4.5.3; R Foundation for Statistical Computing, Vienna, Austria) by two analysts, with identical results. Consequently, the choice of retained row did not influence any laboratory value or outcome used in the analyses. The complete processing code, from the raw extract to all reported estimates, is provided as Supplementary File S1.

### 2.3. Data Collection and Variables

A prespecified sampling window relative to the recorded application date could not be independently verified because sufficiently granular specimen-collection timestamps were not retained in the analytic extract. Consequently, although the analysis used the CRP and albumin measurements linked to each index-encounter record, which were identical across all same-patient/same-date duplicate rows (Section 2.2), their temporal relationship to treatment initiation, evolving organ dysfunction, or clinical deterioration could not be established. These measurements should therefore be interpreted as encounter-associated laboratory values rather than uniformly verified pretreatment baseline biomarkers.

Data were extracted from the hospital laboratory information system and administrative admission and discharge records. For each encounter, the available variables included age at diagnosis, sex, presentation/admission information, serum CRP, serum albumin, serum urea, serum creatinine, hospital admission status, length of stay, discharge date, and death date when available. Laboratory values used in the primary biomarker analyses were the first available measurements linked to the retained index-encounter record, rather than intentionally selected later inpatient measurements; these values were identical across all same-patient/same-date duplicate rows. When both CRP and albumin were available, CAR was derived from those index values. The analytic extract did not retain sufficiently granular sampling-time information to establish that CRP and albumin had been obtained in the same venipuncture, so no such assumption was made. Albumin values below 6 were considered to have been recorded in g/dL and were multiplied by 10 to convert them to g/L; two zero albumin results and non-numeric assay-limit entries (e.g., “<0.10”) or hemolysis entries were treated as missing. All numeric CRP values retained in the cleaned analytic dataset were positive, so log2 transformation did not require zero-value substitution. CAR was calculated throughout as CRP in mg/L divided by albumin in g/L. Numerical CAR values on this scale are one tenth of values calculated with albumin expressed in g/dL; therefore, thresholds should not be compared across studies without accounting for the albumin unit convention. Information on baseline renal function, KDIGO AKI stage, detailed comorbidities, vital signs, infection severity, intensive care admission, medications, treatment limitations, and standardized illness-severity scores was unavailable. Assay-platform and reagent metadata were not retained in the analytic extract, precluding assessment of possible analyzer or method changes over the 2016–2025 study period.

### 2.4. Outcomes

The primary outcome was death recorded in the hospital information system within 30 days of the index encounter. Hospital-recorded death within 180 days was a supportive exploratory outcome. Outcome classification was encounter-specific: for patients with more than one eligible encounter, time from each index date to the recorded death date was evaluated separately, so a single eventual death could satisfy the endpoint for more than one preceding encounter. Because repeated patient-date records were not systematically classified according to whether they belonged to the same hospitalization or separate admissions, the encounter-specific outcome definition should not be interpreted as an admission-specific or independent AKI episode-specific mortality endpoint. Mortality ascertainment was limited to hospital records because linkage to a national or regional death registry was unavailable. The analysis did not separately classify hospital-recorded deaths according to whether they occurred during the index admission or a subsequent hospital encounter. Therefore, the hospital-recorded endpoint indicates that a death date was documented in the hospital information system within the specified interval, but does not establish the admission during which the death occurred. No deaths from external sources were independently ascertained. Data extraction closed on 31 December 2025; therefore, encounters after 1 December 2025 did not have a complete 30-day potential observation window and encounters after 4 July 2025 did not have a complete 180-day potential observation window. The principal analyses retained the defined encounter cohort, while additional date-restricted sensitivity analyses excluded encounters lacking the full potential follow-up window to assess whether administrative closure of the dataset materially affected the findings. In the CAR-complete sample, these date-restricted analyses included 6566 encounters for 30 days and 6110 encounters for 180 days. These analyses address incomplete potential observation time near the end of data collection but cannot recover deaths occurring outside the hospital information system.

For encounters not resulting in admission, a hospital-recorded death within the subsequent 30 days could still be identified when a death date was present in the hospital information system. However, the analytic extract did not retain the provenance of individual death-date entries, preventing verification of whether these records originated from subsequent hospital admissions, later administrative updates, or other internal recording pathways. Accordingly, the absence of a hospital-recorded death within the specified interval was classified as no recorded event rather than independently confirmed survival.

### 2.5. Statistical Analysis

The initial descriptive analyses, primary age- and sex-adjusted logistic models, linearity assessments, and single-marker ROC analyses were performed in R (version 4.5.3). For this revision, the complete workflow—from the raw source extract through deduplication, cleaning, primary and sensitivity analyses, paired DeLong comparisons, and bootstrap procedures—was re-implemented as a single documented script in Python (version 3.12.3) using NumPy (version 2.4.4), pandas (version 3.0.2), SciPy (version 1.17.1), and scikit-learn (version 1.8.0), which reproduced the previously reported estimates. All estimates reported in this manuscript, except the multiple-imputation analysis (performed in R with the mice package, version 3.19.0), were generated or verified with this script (Supplementary File S1); the deduplication audit, first-encounter analysis, patient-level bootstrap, and flexible albumin models were additionally reproduced independently in R by a second analyst, with concordant results. Data types, coding, and missing observations were checked before analysis. Age at diagnosis was used as the age variable throughout. Categorical variables were summarized as *n* (%), while continuous variables were summarized according to their distributions. Distributional form was assessed primarily by graphical inspection; markedly skewed variables were reported as median and interquartile range [median (IQR)] rather than relying on normality-test significance in this large sample.

The Wilcoxon rank-sum test was used for two-group comparisons of non-normally distributed continuous variables, and Pearson’s chi-square test was used for categorical variables where expected counts were adequate; for the 2 × 2 missingness comparison reported below, Yates’ continuity correction was applied. Univariable and multivariable logistic regression models were fitted for binary outcomes, with results reported as odds ratios (OR), 95% confidence intervals (CI), and *p*-values. Age and sex were selected a priori as the consistently available adjustment variables. Because clinical severity and comorbidity data were unavailable, these estimates should not be interpreted as independent of those factors. The functional form of continuous predictors was evaluated by comparing a linear term with a three-degree-of-freedom natural spline [ns(x, df = 3)] using a likelihood-ratio test. CAR and CRP were evaluated on the log2 scale when used as continuous predictors; thus, their ORs correspond to a doubling. Albumin showed evidence of non-linearity in the common-marker sample and was therefore represented flexibly in adjusted discrimination models rather than summarized by a single linear OR. This modeling strategy was intended to avoid forcing a single linear effect estimate onto a marker for which the observed association was not adequately represented by linearity.

Missing data were quantified for each variable. Primary association analyses used complete cases. CAR was unavailable in 578 encounters (8.0%), and the relationship between CAR missingness and the hospital-recorded 30-day outcome was examined. The complete-case approach was used to ensure that CAR, CRP, and albumin were compared within the same observations, avoiding differences in sample composition as an additional source of variation in their estimated discriminatory performance. Because CAR is mathematically derived from CRP and albumin, imputation of CAR as an independent variable would not have been appropriate. The primary analyses were therefore based on observed biomarker values. As a sensitivity analysis for the primary association, multiple imputation by chained equations with predictive mean matching was performed in all 7181 encounters: missing CRP (*n* = 285), albumin (*n* = 404), and creatinine (*n* = 111) values were imputed separately, with age, sex, and 30-day mortality (all complete) included in the imputation model; CAR was not imputed directly but was recalculated from the imputed components within each completed dataset. Thirty imputed datasets were generated (20 iterations), and estimates were pooled using Rubin’s rules. The associations and discrimination measures were also examined across patient-clustered, first-encounter, date-restricted, and hospitalized-only sensitivity analyses. These analyses assessed the robustness of the findings to several cohort and outcome-definition choices but were not designed to correct selection bias arising from missing biomarker data.

Receiver operating characteristic (ROC) analysis was used to assess discrimination. For the principal component comparison, albumin, CRP, and CAR were evaluated in the identical complete-marker sample (*n* = 6603), and AUCs with 95% CIs were estimated using the DeLong method [27]. For the exploratory 180-day endpoint, the descriptive standalone AUC for albumin was also calculated on the same complete-marker sample. Formal between-marker comparisons used paired DeLong tests. Because these paired tests are performed at the encounter level and do not account for repeated observations from the same patient, uncertainty in the AUC differences was additionally estimated by patient-level bootstrap resampling (described below), and the comparisons were repeated in the first-encounter sample, in which each patient contributed a single observation. To examine whether the ratio added useful information beyond routinely available covariates, apparent discrimination was also estimated for a base model containing age and sex and for models adding log2(CAR), log2(CRP), flexible albumin, or flexible albumin plus log2(CRP). Flexible albumin was represented by a natural cubic spline with three degrees of freedom (boundary knots at the minimum and maximum, interior knots at the tertiles). An additional structural comparison entered log2(CRP) and log2(albumin) as separate linear terms, thereby relaxing the equal-magnitude, opposite-direction coefficient constraint implicit in a single log2(CAR) term. These models were evaluated in the same *n* = 6603 sample using AUC, Akaike information criterion (AIC), and Brier score; because model development and evaluation used the same cohort, these values represent apparent performance, and optimism-corrected estimates were obtained by bootstrap as described below. Internal validation and uncertainty in AUC differences were assessed by patient-level (cluster) bootstrap resampling with 2000 resamples: in each resample, patients rather than encounters were sampled with replacement, and all encounters of each selected patient were retained. Percentile 95% CIs were obtained for the differences in standalone AUC (albumin vs. CAR, albumin vs. CRP, and CAR vs. CRP) and for the differences in apparent AUC between adjusted models. Optimism was estimated with Harrell’s bootstrap procedure: each model, including re-estimation of spline knots, was refitted in every bootstrap sample and evaluated both in that sample and in the original sample, and the mean difference was subtracted from the apparent AUC to obtain the optimism-corrected AUC. Calibration was not evaluated, as the primary objective was the relative comparison of biomarkers rather than the development of an individual clinical prediction tool.

Repeated encounters were addressed in two complementary ways. First, the age- and sex-adjusted CAR models were refitted with standard errors clustered by patient identifier, preserving all eligible encounters while accounting for within-patient correlation in regression inference. Second, the previously specified sensitivity analysis restricted the data to each patient’s chronologically first eligible encounter (5744 encounters, of which 5420 had complete CRP and albumin values), thereby removing repeated encounter-level attribution and within-patient dependence; in this sample, adjusted odds ratios, standalone AUCs with 95% CIs, and paired DeLong comparisons were estimated for both the 30-day and the exploratory 180-day outcomes. In addition, date-restricted analyses excluded encounters without complete potential 30- or 180-day follow-up. Because mortality ascertainment was limited to hospital records and might differ after emergency-department discharge, an additional sensitivity analysis restricted the common-marker sample to encounters resulting in hospital admission; this analysis repeated the age- and sex-adjusted CAR model and same-sample marker ROC comparison. Cluster-robust inference does not change regression point estimates, but it provides more appropriate confidence intervals when observations from the same patient are correlated. The first-encounter analysis remained important because clustering alone does not prevent one recorded death from satisfying the endpoint for several earlier encounters, and standard paired DeLong inference does not explicitly model patient-level clustering; the latter was additionally addressed by the patient-level bootstrap. For transparency, the sample sizes, numbers of hospital-recorded deaths, age- and sex-adjusted CAR odds ratios with 95% confidence intervals, and available discrimination estimates from the primary and sensitivity analyses are summarized in the Results section. Discrimination measures were not recalculated for the date-restricted subsets; patient-clustered regression inference applies to the odds ratios, whereas cluster-aware uncertainty for the AUC differences was obtained from the patient-level bootstrap.

**Figure 2:**
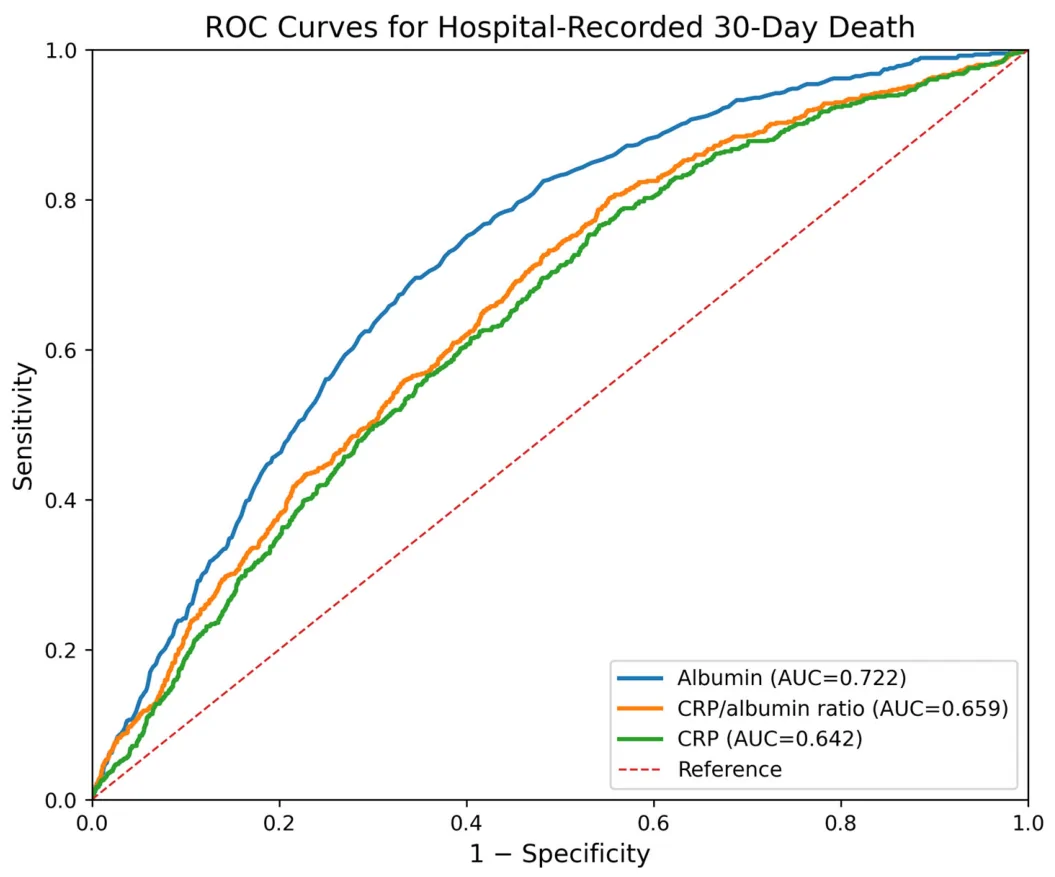
Receiver operating characteristic curves for hospital-recorded 30-day death using albumin, CAR calculated as CRP (mg/L) divided by albumin (g/L), and CRP in the identical complete-marker sample (n = 6603). Lower albumin values were oriented toward higher risk. All formal between-marker comparisons therefore use the same observations.

All tests were two-sided and *p* < 0.05 was considered statistically significant. Very small *p*-values are reported as *p* < 0.001. No multiplicity adjustment was applied to secondary or exploratory analyses; these results should therefore be interpreted as descriptive and hypothesis-generating. Data management and the reported R analyses used dplyr (version 1.2.1), broom (version 1.0.13), pROC (version 1.19.0.1), splines (version 4.5.3), mice (version 3.19.0), and ggplot2 (version 4.0.3); the Python workflow (Supplementary File S1) used the Python packages listed above and matplotlib (version 3.10.8) for Figure 1 and Figure 2.

## 3. Results

### 3.1. Baseline Characteristics

A total of 7181 patient-date encounters with an ICD-10-coded diagnosis of AKI were included, representing 5744 unique patients; 1048 patients contributed more than one eligible encounter. Of the 7181 encounters, 3994 (55.6%) resulted in hospitalization and 3187 (44.4%) did not. Age and sex were available for all encounters. CAR was available for 6603 encounters (92.0%). The principal characteristics and biomarker distributions are summarized in Table 1.

Among the 6603 encounters with available CAR measurements, 658 (10.0%) had a hospital-recorded death within 30 days, compared with 29 of 578 encounters (5.0%) with missing CAR. This difference indicates that the availability of complete biomarker measurements was associated with the observed outcome and supports the possibility of selection bias in complete-case analyses. However, the available comparisons do not establish the underlying missing-data mechanism.

### 3.2. CRP/Albumin Ratio and Hospital-Recorded 30-Day Death

A death within 30 days was recorded for 687 of 7181 encounters (9.6%). CAR data were available for 6603 encounters (92.0%); 578 encounters (8.0%) had missing CAR. CAR missingness was associated with the recorded 30-day outcome (chi-square = 14.47, df = 1, *p* < 0.001), indicating that complete-case estimates could be affected by outcome-associated missingness and should be interpreted alongside the other robustness analyses. Because the raw 9.6% proportion includes a small number of late-2025 encounters without a complete potential 30-day observation window, it should be interpreted as the observed hospital-recorded event proportion rather than a registry-based population mortality estimate.

CAR was higher in encounters with death recorded within 30 days than in encounters without recorded 30-day death (median [IQR] 2.32 [0.659–6.42] vs. 0.690 [0.175–3.09]; Wilcoxon rank-sum *p* < 0.001). On the raw scale, the association was non-linear (likelihood-ratio chi-square = 54.16, df = 2, *p* < 0.001). After log2 transformation, no significant residual non-linearity was detected (chi-square = 1.93, df = 2, *p* = 0.381), so subsequent ORs are reported per doubling of CAR.

After adjustment for age and sex, each doubling of CAR was associated with approximately 25% higher odds of a hospital-recorded death within 30 days (adjusted OR 1.25, 95% CI 1.20–1.29, *p* < 0.001). Patient-clustered standard errors left the conclusion unchanged (OR 1.246, 95% CI 1.197–1.296, *p* < 0.001; 6603 encounters from 5456 patients). When encounters without a complete potential 30-day follow-up window were excluded, 6566 CAR-complete encounters remained (655 recorded deaths); the clustered estimate was again essentially unchanged (OR 1.247, 95% CI 1.199–1.298, *p* < 0.001). In the multiple-imputation sensitivity analysis including all 7181 encounters (687 recorded deaths), the pooled estimate was very similar (OR 1.254, 95% CI 1.210–1.299, *p* < 0.001).

CAR showed modest standalone discrimination for recorded 30-day death (AUC 0.659, 95% CI 0.638–0.680). Because the study was designed as a comparative biomarker analysis rather than to derive a clinical decision rule, no sample-derived CAR cut-off was interpreted as clinically actionable.

### 3.3. Exploratory CRP/Albumin Ratio and Hospital-Recorded 180-Day Death

A death within 180 days was recorded for 942 of 7181 encounters (13.1%). In the CAR-complete sample (*n* = 6603), 893 encounters met this recorded endpoint. CAR was higher among encounters with a recorded death within 180 days than among encounters without such a death (median [IQR] 1.98 [0.545–5.76] vs. 0.677 [0.172–3.02]; *p* < 0.001). After log2 transformation, no significant residual non-linearity was detected (chi-square = 4.28, df = 2, *p* = 0.117).

In the age- and sex-adjusted model, each doubling of CAR was associated with higher odds of a hospital-recorded death within 180 days (adjusted OR 1.19, 95% CI 1.16–1.23, *p* < 0.001). Clustered inference by patient was similar (OR 1.190, 95% CI 1.151–1.231, *p* < 0.001). Restricting the analysis to the 6110 CAR-complete encounters with a full potential 180-day observation window left the clustered estimate materially unchanged (OR 1.188, 95% CI 1.148–1.230, *p* < 0.001; 846 recorded deaths). In the complete-marker sample, albumin discriminated the exploratory 180-day endpoint with an AUC of 0.701 (CAR AUC 0.633).

Standalone discrimination for the recorded 180-day outcome remained modest (AUC 0.633, 95% CI 0.613–0.652). Despite the reassuring date-restricted sensitivity analysis, the 180-day result remains supportive and exploratory because mortality ascertainment was limited to hospital records and a single eventual death could be attributed to more than one preceding encounter.

### 3.4. CRP/Albumin Ratio Versus Its Individual Components

To make the biomarker comparison directly interpretable, albumin, CRP, and CAR were evaluated on the identical complete-marker sample of 6603 encounters (658 recorded 30-day deaths). Standalone AUCs were 0.722 (95% CI 0.703–0.740) for albumin, 0.659 (95% CI 0.638–0.680) for CAR, and 0.642 (95% CI 0.620–0.663) for CRP. Albumin discriminated the outcome better than CAR (paired DeLong z = 5.83, *p* < 0.001) and better than CRP (z = 6.86, *p* < 0.001). CAR also discriminated the outcome better than CRP in paired DeLong testing (*p* < 0.001), although the absolute AUC difference was small (0.018, calculated from unrounded estimates). The corresponding ROC curves are shown in Figure 2, and the AUC estimates are summarized in Table 2.

These same-sample comparisons show that the ratio did not improve standalone discrimination over albumin. Because biomarker-only ROC curves do not address incremental value beyond clinical covariates, adjusted model comparisons were also performed as described in Section 3.6. Patient-level bootstrap resampling, which accounts for repeated encounters from the same patient, gave 95% CIs for the AUC differences of 0.040–0.085 for albumin versus CAR (difference 0.062), 0.056–0.105 for albumin versus CRP (0.080), and 0.015–0.021 for CAR versus CRP (0.018); the direction of these differences was not reversed in any bootstrap resample.

### 3.5. Sensitivity Analyses: First Eligible Encounter and Hospitalized Encounters

The analysis restricted to the chronologically first eligible encounter for each of 5744 unique patients yielded materially unchanged principal findings. In the common complete-marker subset of first encounters (*n* = 5420; 559 hospital-recorded 30-day deaths), each patient contributed a single observation. The age- and sex-adjusted association between CAR and recorded 30-day death was similar to the primary encounter-level estimate (adjusted OR per doubling 1.225, 95% CI 1.180–1.271). Standalone AUCs were 0.716 (95% CI 0.695–0.737) for albumin, 0.651 (0.628–0.674) for CAR, and 0.634 (0.611–0.657) for CRP; albumin discriminated better than CAR (difference 0.065, 95% CI 0.042–0.088; paired DeLong *p* < 0.001) and CRP (difference 0.082, 95% CI 0.057–0.107; *p* < 0.001), and CAR slightly better than CRP (difference 0.017, 95% CI 0.014–0.020; *p* < 0.001). For the exploratory 180-day outcome (751 deaths), the adjusted OR per doubling of CAR was 1.174 (95% CI 1.137–1.212), and AUCs were 0.700 (0.681–0.718) for albumin, 0.625 (0.604–0.646) for CAR, and 0.609 (0.588–0.630) for CRP (albumin vs. CAR difference 0.075, 95% CI 0.055–0.096; *p* < 0.001). A separate audit identified 159 deceased patients for whom the same recorded death date was linked to more than one eligible encounter. The concordance of the first-encounter, patient-clustered, and patient-level bootstrap analyses supports the robustness of the principal 30-day association and marker ranking, while also showing why encounter-level mortality attribution should be interpreted cautiously.

As a separate outcome-ascertainment sensitivity analysis, the cohort was restricted to hospitalized encounters. Among 3695 hospitalized encounters with complete albumin, CRP, and CAR data, 453 hospital-recorded 30-day deaths occurred. The age- and sex-adjusted association between CAR and recorded 30-day death remained present (OR per doubling 1.16, 95% CI 1.11–1.21, *p* < 0.001). Albumin again discriminated the outcome better than CAR (AUC 0.693 vs. 0.606; paired DeLong z = 6.34, *p* < 0.001), while CRP had an AUC of 0.588. Restricting the analysis to encounters resulting in hospital admission therefore did not alter the principal biomarker ranking. A consolidated comparison of the primary and sensitivity analyses, including available sample sizes, hospital-recorded event counts, adjusted odds ratios, confidence intervals, and discrimination measures, is provided in Section 3.6.

Compared with the primary analysis, the hospitalized-only association was attenuated (adjusted OR 1.16 vs. 1.246 with patient-clustered inference), as was CAR discrimination (AUC 0.606 vs. 0.659). Among the 2908 encounters with complete markers that did not result in hospitalization, 205 (7.0%) had a hospital-recorded death date within 30 days. These death dates were documented in the hospital information system, but their recording pathways and the locations of death could not be independently verified.

### 3.6. Age- and Sex-Adjusted Marker Comparison

Apparent performance of the age- and sex-adjusted models is summarized in Table 3; a consolidated summary of the primary and sensitivity analyses is given in Table 4, and patient-level bootstrap estimates of the differences are shown in Table 5.

Albumin was modeled flexibly because of a non-linear association with recorded 30-day death (likelihood-ratio chi-square = 28.64, df = 2, *p* < 0.001, versus a linear albumin term in the age- and sex-adjusted model). The flexible albumin model discriminated better than the CAR-containing model (AUC 0.734 vs. 0.688; difference 0.045, bootstrap 95% CI 0.027–0.065; paired DeLong *p* < 0.001). Entering log2(CRP) and log2(albumin) as separate terms yielded a higher apparent AUC than CAR alone (0.737, 95% CI 0.719–0.755, vs. 0.688; difference 0.049, bootstrap 95% CI 0.034–0.065; paired DeLong *p* < 0.001). Adding log2(CRP) to flexible albumin increased AUC by only 0.006 (0.734 to 0.740; bootstrap 95% CI 0.002–0.013), a small difference of uncertain clinical importance. Optimism estimated by the patient-level bootstrap was small for all models (0.001–0.002), yielding optimism-corrected AUCs of 0.608 (age + sex), 0.687 (+log2[CAR]), 0.674 (+log2[CRP]), 0.736 (+log2[CRP] + log2[albumin]), 0.732 (+flexible albumin), and 0.738 (+flexible albumin + log2[CRP]); the optimism-corrected differences between the component-based models and the CAR model (0.046 and 0.049) were essentially identical to the apparent differences. All model comparisons remained exploratory, without calibration assessment or external validation.

## 4. Discussion

This study evaluated CAR and its individual components in a large hospital-based cohort of N17-coded AKI encounters without recorded N18.x CKD. Higher CAR was associated with hospital-recorded 30-day death after adjustment for age and sex, with similar estimates in patient-clustered, first-encounter, and date-restricted analyses. However, restricting the analysis to hospitalized encounters resulted in an attenuated association and lower discriminatory performance, although albumin continued to outperform CAR. These findings support consistency in the direction of association and relative biomarker ranking, but not complete stability of the estimates across care settings. The more informative finding, however, was comparative: albumin alone discriminated the 30-day outcome better than CAR in the identical observations, and the same hierarchy persisted in the hospitalized-only analysis. Modeling albumin and CRP as separate components also produced greater apparent discrimination than the ratio, both in the explicit log-component comparison and when albumin was represented flexibly. These results suggest that CAR carries prognostic signal in code-identified AKI but that, in this cohort, the ratio provided lower apparent discrimination than approaches retaining albumin and CRP as separate predictors. This negative comparative finding is itself clinically relevant: a conveniently calculated composite biomarker should not be assumed to improve risk stratification merely because each of its components carries prognostic information.

In the common complete-marker sample, standalone AUC was 0.722 for albumin versus 0.659 for CAR. When added to age and sex, a model retaining CRP and albumin as separate log-transformed predictors achieved greater apparent discrimination than a model containing CAR (AUC 0.737 vs. 0.688). These differences were robust to patient-level bootstrap resampling and showed negligible optimism, but they require independent external validation before the biomarker ranking can be generalized to other populations.

The present findings add an important counterpoint to prior CAR literature. Park et al. reported that CAR modestly outperformed CRP for 28-day mortality in critically ill patients [10], Ranzani et al. demonstrated prognostic value for CAR at ICU discharge in sepsis [11], and Wang et al. reported an association between CAR and mortality in critically ill AKI [12]. Jeon et al. studied 3995 patients with severe AKI requiring continuous renal replacement therapy across eight centers and found that higher CAR was associated with in-hospital mortality; adding CAR to APACHE II or SOFA scores produced modest improvements in discrimination and reclassification [13]. In contrast, Liu and Lv analyzed 358 ICU patients with AKI from the Medical Information Mart for Intensive Care III (MIMIC-III) database and reported AUCs of approximately 0.64 for CAR, 0.63 for CRP, and 0.59 for albumin for 365-day mortality [14], whereas in the present common-marker sample albumin had the highest AUC (0.722), followed by CAR (0.659) and CRP (0.642). A 2026 cohort from the Foggia group likewise reported an association between CAR and short-term in-hospital mortality across adult age groups [28]. Differences in case mix, AKI definition and severity, timing of biomarker measurement, follow-up horizon, and prevalence of sepsis or other inflammatory conditions probably contribute to the variation in relative marker performance across studies. The practical implication is that the prognostic value of CAR should be demonstrated against its individual components in the target population rather than assumed from results obtained in a different clinical setting.

The stronger performance of albumin in this cohort is biologically plausible. Albumin is not simply a nutritional marker or a passive denominator in CAR; during acute illness it reflects inflammation, endothelial permeability and capillary leak, redistribution between intravascular and extravascular compartments, hepatic synthesis, losses, extracellular volume, and pre-existing disease [7,8]. Its association with adverse outcomes in AKI is supported by prior meta-analytic evidence [9]. In septic AKI treated with continuous renal replacement therapy, higher albumin has been associated with lower mortality after multivariable adjustment [29]. CAR has also shown prognostic value in hospitalized COVID-19 populations [30] and in incident hemodialysis cohorts [31], but these populations differ substantially from the present broad hospital cohort. The non-linear albumin association observed here is particularly relevant: it suggests that forcing albumin into the denominator of a fixed ratio may oversimplify a relationship whose prognostic effect changes across the concentration range.

The characteristics of the study population may also have contributed to the observed relative performance of albumin and CAR. In particular, patients with documented CKD were excluded at the source-extraction stage using ICD-10 N18.x codes. Because CKD may influence circulating albumin concentrations through inflammation, protein losses, fluid balance, and associated comorbidity, excluding patients with recognized CKD may have altered the distributions and prognostic relationships of albumin and CRP within the analyzed cohort. This selection may therefore have influenced the relative discriminatory performance of albumin, CRP, and their ratio. However, the direction and magnitude of this potential effect cannot be established from the available data, particularly because unrecognized or uncoded CKD could not be excluded. The findings should consequently be interpreted as specific to this administratively selected population rather than assumed to apply to patients with AKI superimposed on CKD or to unselected AKI populations.

The statistical behavior of a ratio offers a second explanation for the findings. Because log(CAR) equals log(CRP) minus log(albumin), a single coefficient for log(CAR) constrains the two components to contribute with equal magnitude and opposite direction. That constraint is unlikely to be optimal when albumin has a stronger, non-linear association with outcome while CRP contributes a weaker inflammatory signal. More generally, indiscriminate use of ratios can create interpretive and modeling problems when the denominator has an independent or nonlinear relation with the outcome [20,21]. Recent methodological work similarly notes that combining variables into a ratio can prevent separation of component-specific information and can impose assumptions that should be justified by the scientific question, even when ratios remain convenient for prognostic or predictive use [21]. Complementary simulation evidence indicates that omission of the component main effects in ratio-centered regression can distort inference; although that work was developed chiefly for causal questions, it further illustrates why direct component modeling is an important comparator when evaluating a prognostic ratio [22]. The explicit log-component model in the present study supported this interpretation: entering log2(CRP) and log2(albumin) separately increased apparent AUC from 0.688 for the CAR-containing model to 0.737, while the model using flexible albumin plus log2(CRP) reached 0.740. These comparisons were internally validated by bootstrap but do not establish an externally validated prediction model; rather, they are consistent with greater apparent discrimination when the components are allowed to contribute independently instead of being constrained to a fixed ratio. This interpretation also accords with contemporary biomarker guidance: the Acute Disease Quality Initiative recommends combining biomarker information with functional measures and clinical data rather than using a biomarker as a stand-alone decision rule [32]. A systematic review and meta-analysis in sepsis supports prognostic signal for CAR but also demonstrates substantial between-study heterogeneity [33], and contemporary ADQI guidance for sepsis-associated AKI similarly emphasizes mechanistic heterogeneity and context-specific phenotyping [34]. Future work should therefore focus less on identifying a universal CAR threshold and more on whether CRP, albumin, their trajectories, and other clinical variables provide reproducible incremental information, adequate calibration, and clinically useful net benefit. Importantly, although retaining CRP and albumin as separate predictors improved apparent discrimination compared with the CAR-containing model, the additional contribution of CRP beyond a model containing flexibly modeled albumin was small (ΔAUC = 0.006). Such a modest difference in apparent discrimination does not necessarily translate into meaningful improvement in clinical risk assessment, treatment decisions, or patient outcomes. Without external validation, calibration assessment, and decision-curve analysis, the present findings support comparisons of biomarker performance rather than claims of improved clinical decision-making.

From a clinical perspective, these findings do not support replacing albumin with CAR or using CAR as a treatment or disposition rule. The component-level results instead suggest that clinically useful prognostic models should retain CRP and albumin as separate variables unless a ratio demonstrates reproducible incremental performance after appropriate clinical adjustment and validation. Because the present study did not include a fully specified severity model, external validation, calibration assessment, or decision-analytic evaluation, the findings should be interpreted as comparative biomarker evidence rather than as a new clinical risk score.

The study has several strengths. It includes 7181 encounters representing 5744 unique patients collected over a decade and therefore evaluates a broader code-identified hospital population than many prior ICU-focused CAR studies, while still excluding patients with recorded CKD at source extraction. The principal biomarker comparison was performed in the same complete-marker sample, avoiding the misleading comparison of AUCs obtained from different subsets. Continuous predictors were examined for non-linearity; CAR and CRP were analyzed on a log2 scale; albumin was modeled flexibly when appropriate; and adjusted models were compared using AUC, AIC, and Brier score rather than discrimination alone. The ratio constraint was also tested directly by entering log2(CRP) and log2(albumin) as separate terms. The main CAR association was additionally examined with patient-clustered inference, a one-encounter-per-patient analysis, date-restricted complete-potential-follow-up analyses, a hospitalized-only sensitivity analysis addressing differential outcome ascertainment by care pathway, and patient-level bootstrap resampling with optimism correction for the discrimination comparisons. The deduplication and cleaning procedure was reconstructed from the raw source extract, and the complete analytic workflow is provided as reproducible code. Collectively, these features strengthen the central inference that CAR is associated with risk but did not outperform albumin in this cohort.

Several limitations remain and should define the boundaries of interpretation. First, this was a retrospective, single-center study based on routinely collected data. AKI was identified using ICD-10 codes N17.x and could not be independently confirmed or staged using baseline creatinine and urine-output criteria; the results therefore apply to clinically coded AKI encounters rather than to all laboratory-defined AKI. Validation studies indicate that coded AKI can have relatively high positive predictive value but limited sensitivity, so code-identified cohorts may preferentially capture clinically recognized disease [23,24]. This administrative case definition may have introduced selection bias by preferentially capturing encounters in which AKI was clinically recognized and explicitly coded, while potentially missing milder, transient, or otherwise uncoded episodes that would have fulfilled KDIGO criteria. Consequently, the study population may differ from an unselected, laboratory-defined AKI cohort in terms of disease severity, clinical characteristics, and mortality risk. Because KDIGO-defined AKI could not be independently ascertained, the magnitude and direction of this selection bias could not be quantified. These differences may also influence the observed associations and relative discriminatory performance of CAR and its individual components. Therefore, the findings should not be generalized without further validation to all patients meeting KDIGO criteria, particularly those with less severe or unrecognized AKI. Chronic kidney disease was excluded administratively at the initial database-extraction stage using recorded N18.x codes, but unrecognized or uncoded CKD and maintenance dialysis could not be completely excluded. Conversely, the deliberate exclusion of recognized CKD means that the cohort does not represent the full spectrum of AKI, particularly AKI superimposed on established CKD; this may have altered case mix, baseline albumin distributions, and absolute mortality risk and limits generalizability to patients with known CKD. No representative medical-record validation substudy was performed to determine the proportion of coded encounters fulfilling KDIGO criteria or to identify potentially misclassified cases of AKI superimposed on CKD and patients receiving maintenance dialysis. Consequently, the extent of these potential classification errors remains unknown. Second, detailed comorbidities, baseline renal function, infection source, vital signs, ICU admission, medications, treatment limitations, and standardized severity scores were unavailable. Age- and sex-adjusted estimates therefore should not be interpreted as independent of clinical severity or comorbidity, and the study could not construct a fully specified clinical risk model against which incremental biomarker value might be judged. Furthermore, albumin and CAR may reflect several overlapping clinical processes, including infection, hepatic dysfunction, malignancy, fluid imbalance, and overall illness severity. Because these factors were not systematically available in the analytic dataset, residual confounding cannot be excluded. The observed associations may therefore partly reflect underlying clinical vulnerability rather than biomarker-specific prognostic information. No additional medical-record-based subgroup analysis incorporating these clinical variables was performed, and the extent to which their inclusion might have altered the estimated associations or comparative discrimination remains unknown. Future studies should assess the incremental prognostic value of CAR and its components after adjustment for clinically relevant comorbidities and validated measures of illness severity. Creatinine and urea were available, but without baseline kidney function or adjudicated AKI stage they could not substitute for a comprehensive severity phenotype. Assay-platform and reagent metadata were not retained in the analytic extract, so possible laboratory-method changes across the decade-long study period could not be quantified.

Repeated encounters and outcome ascertainment introduce additional uncertainty. Although patient-clustered standard errors account for within-patient correlation when estimating regression uncertainty, they do not alter the regression point estimates or eliminate potential bias arising from repeated encounter-level attribution of the same eventual death. Consequently, patients with multiple eligible encounters may contribute disproportionately to the encounter-level analyses, and these observations should not be interpreted as independent AKI episodes. Furthermore, the inability to reliably distinguish new admissions from follow-up encounters or repeated assessments within the same hospitalization introduces additional uncertainty regarding the clinical independence of patient-date observations and their interpretation as distinct episodes of care. The first-encounter sensitivity analysis mitigated this concern and reproduced both the principal association between CAR and hospital-recorded 30-day mortality and the superior discrimination of albumin over CAR. In addition, patient-level bootstrap resampling, which preserves within-patient dependence, yielded confidence intervals for the AUC differences similar to the encounter-level DeLong intervals, and these excluded zero for all principal comparisons. Mortality ascertainment was limited to the hospital information system without linkage to a national or regional death registry, so deaths occurring elsewhere could have been missed, particularly after discharge from the emergency department or hospital. Consequently, the reported 30-day and 180-day hospital-recorded mortality proportions may underestimate all-cause mortality in the underlying cohort. Deaths occurring outside the hospital information system could have been incorrectly classified as the absence of a recorded death, and the extent of this potential under-ascertainment could not be quantified. Such outcome misclassification may also have affected the estimated associations and discriminatory performance of the evaluated biomarkers, although its direction and magnitude remain uncertain. Because the cohort included both hospitalized and non-hospitalized encounters, completeness of outcome capture may also have differed according to subsequent care pathway. The hospitalized-only sensitivity analysis reproduced the association and biomarker ranking, but it cannot recover deaths occurring outside the hospital system after discharge and therefore only partially addresses this limitation. Date-restricted analyses address incomplete potential follow-up near the end of data collection but cannot correct external deaths that were never recorded. The decade-long study period also spans possible changes in coding, testing, treatment practice, and the COVID-19 era, while calendar-time effects were not explicitly modeled. Finally, CAR was unavailable in approximately 8% of encounters, and missingness was significantly associated with hospital-recorded 30-day death. Consequently, the assumption that encounters with complete biomarker measurements were representative of the entire eligible cohort cannot be established. Missing CRP or albumin measurements may have reflected clinical testing decisions, care pathways, or other patient characteristics associated with prognosis, although the underlying reasons for missingness could not be determined from the available data. The complete-case strategy enabled direct comparisons of CAR and its individual components within an identical sample but did not eliminate the possibility of selection bias. A multiple-imputation sensitivity analysis yielded an association very similar to the complete-case estimate; however, it assumes that missingness is at random conditional on the imputation-model variables, and assay-limit CRP entries (e.g., “<0.10”), which indicate very low values, were treated as missing and imputed. Imputation was applied to the primary association only; the AUCs and the relative marker ranking were not re-estimated in imputed data, and the influence of missing biomarker data on these comparisons remains uncertain. Although internal validation indicated minimal optimism, all comparisons derive from a single center and have not been externally validated; external, registry-linked validation is required before any CAR-based decision threshold or treatment application is proposed. Furthermore, model calibration was not assessed; therefore, the agreement between predicted and observed event probabilities remains unknown. No additional analysis adjusting for calendar year or stratifying the cohort into pre-pandemic, pandemic, and post-pandemic periods was performed. Therefore, we could not determine whether the associations between CAR, albumin, and hospital-recorded mortality or their relative discriminatory performance remained stable throughout the study period. Potential temporal changes in patient characteristics, diagnostic coding, laboratory procedures, and clinical management may have influenced the observed associations. This limitation should be considered when interpreting the pooled estimates obtained across the entire decade.

## 5. Conclusions

In conclusion, among hospital encounters with an N17-coded diagnosis of AKI after source-level exclusion of recorded N18.x CKD, higher CAR was associated with hospital-recorded 30-day mortality in the primary analysis and several sensitivity analyses. In hospitalized-only encounters, the direction of association persisted, but its magnitude and discrimination were attenuated. Overall, CAR showed only modest standalone discrimination and did not outperform albumin in the same observations. Both the explicit log-component model and the flexible component model yielded greater discrimination than the CAR-containing model, a difference that persisted after patient-level bootstrap resampling and optimism correction. The central comparative finding is therefore not that CAR lacks prognostic association, but that, in this cohort, the ratio showed lower apparent discrimination than approaches retaining its components as separate predictors. The findings support CAR as a descriptive risk marker rather than a stand-alone clinical decision tool and, more broadly, caution against assuming that a biomarker ratio is superior to its components. Importantly, these conclusions are restricted to administratively identified, N17-coded AKI encounters and should not be assumed to apply to the broader population of patients with clinically adjudicated or laboratory-defined AKI. Furthermore, because mortality ascertainment was restricted to the hospital information system, the observed mortality proportions may underestimate overall mortality, and the findings should be interpreted as associations with hospital-recorded rather than comprehensively ascertained all-cause mortality. Future studies should prioritize clinically adjudicated AKI with clearly defined index episodes, registry-linked mortality ascertainment, cluster-aware model evaluation, and external validation in independent cohorts. Until such validation is available, the present findings, particularly those concerning the exploratory 180-day outcome, should not be used to define CAR-based clinical decision thresholds.

## Figures and Tables

**Table 1:** Baseline characteristics of the analytic encounters (*N* = 7181). Biomarker values are shown as median [IQR] in the units used for analysis. CAR was calculated as CRP (mg/L) divided by albumin (g/L). SD, standard deviation; IQR, interquartile range; CRP, C-reactive protein; CAR, C-reactive protein-to-albumin ratio.

Characteristic	Value
Encounters, *n*	7181
Unique patients, *n*	5744
Age at diagnosis, mean ± SD, years	71.20 ± 15.22
Age at diagnosis, median [IQR], years	74 [63–82]
Sex, male, *n* (%)	3819 (53.2%)
Sex, female, *n* (%)	3362 (46.8%)
Hospital admission, *n* (%)	3994 (55.6%)
Hospital-recorded 30-day death, *n* (%)	687 (9.6%)
Hospital-recorded 180-day death, *n* (%)	942 (13.1%)
Albumin available, *n* (%)	6777 (94.4%)
Albumin, median [IQR], g/L	36.0 [31.2–40.2]
CRP available, *n* (%)	6896 (96.0%)
CRP, median [IQR], mg/L	26.28 [7.11–110.02]
CAR available, *n* (%)	6603 (92.0%)
CAR [(mg/L)/(g/L)], median [IQR]	0.784 [0.197–3.481]
Creatinine available, *n* (%)	7070 (98.5%)
Creatinine, median [IQR], mg/dL	3.05 [2.10–5.30]

**Table 2:** Standalone discrimination of hospital-recorded 30-day death by albumin, CAR calculated as CRP (mg/L) divided by albumin (g/L), and CRP in the common complete-marker sample (*n* = 6603). AUC confidence intervals were estimated using the DeLong method.

Predictor	*n*	AUC (95% CI)
Albumin (g/L)	6603	0.722 (0.703–0.740)
CAR (CRP mg/L/albumin g/L)	6603	0.659 (0.638–0.680)
CRP (mg/L)	6603	0.642 (0.620–0.663)

**Table 3:** Apparent discrimination and model fit of age- and sex-adjusted models for hospital-recorded 30-day death in the common complete-marker sample (*n* = 6603). AUCs are in-sample estimates; lower AIC and Brier scores indicate better apparent fit. Flexible albumin: natural cubic spline with 3 degrees of freedom; AIC values for the flexible albumin models were recalculated with the documented spline specification.

Model	AUC (95% CI)	AIC	Brier Score
Age + sex	0.610 (0.588–0.631)	4192.5	0.0885
Age + sex + log2(CAR)	0.688 (0.669–0.708)	4029.1	0.0863
Age + sex + log2(CRP)	0.676 (0.656–0.695)	4064.1	0.0868
Age + sex + log2(CRP) + log2(albumin)	0.737 (0.719–0.755)	3899.5	0.0848
Age + sex + flexible albumin	0.734 (0.715–0.752)	3880.4	0.0844
Age + sex + flexible albumin + log2(CRP)	0.740 (0.722–0.758)	3861.0	0.0840

**Table 4:** Summary of primary and sensitivity analyses for hospital-recorded 30-day mortality and exploratory 180-day mortality. Adjusted ORs refer to each doubling of CAR after adjustment for age and sex.

Analysis	*n*	Deaths	Adjusted OR (95% CI)	AUC CAR/Albumin
30-day: primary complete-marker	6603	658	1.25 (1.20–1.29)	0.659/0.722
30-day: patient-clustered	6603	658	1.246 (1.197–1.296)	0.659 */0.722 *
30-day: date-restricted, clustered	6566	655	1.247 (1.199–1.298)	NR/NR
30-day: first eligible encounter	5420	559	1.225 (1.180–1.271)	0.651/0.716
30-day: multiple imputation (pooled)	7181	687	1.254 (1.210–1.299)	NR/NR
30-day: hospitalized-only	3695	453	1.16 (1.11–1.21)	0.606/0.693
180-day: exploratory complete-marker	6603	893	1.19 (1.16–1.23)	0.633/0.701
180-day: patient-clustered	6603	893	1.190 (1.151–1.231)	0.633 */0.701 *
180-day: date-restricted, clustered	6110	846	1.188 (1.148–1.230)	NR/NR
180-day: first eligible encounter	5420	751	1.174 (1.137–1.212)	0.625/0.700

* Patient-clustered standard errors concern regression only; starred AUCs are unchanged encounter-level estimates (not clustered ROC inference). The 180-day albumin AUC was calculated in the common complete-marker sample. First-encounter analyses include one encounter per patient (complete-marker sample). NR = not calculated (date-restricted subsets and imputed data). Patient-level bootstrap CIs for AUC differences are given in Table 5. Deaths outside the hospital information system may have been missed.

**Table 5:** Patient-level bootstrap estimates of differences in discrimination for hospital-recorded 30-day death in the common complete-marker sample (*n* = 6603 encounters from 5456 patients; 2000 resamples of patients with all of their encounters). Adjusted models include age and sex.

Comparison	AUC Difference	Bootstrap 95% CI
Standalone: albumin—CAR	0.062	0.040–0.085
Standalone: albumin—CRP	0.080	0.056–0.105
Standalone: CAR—CRP	0.018	0.015–0.021
Adjusted: flexible albumin—log2(CAR)	0.045	0.027–0.065
Adjusted: log2(CRP) + log2(albumin)—log2(CAR)	0.049	0.034–0.065
Adjusted: flexible albumin + log2(CRP)—flexible albumin	0.006	0.002–0.013

Differences for adjusted models refer to apparent AUCs; the corresponding optimism-corrected differences were 0.046, 0.049, and 0.006. The direction of the three standalone differences and of the first two adjusted-model differences was not reversed in any bootstrap resample. CI, confidence interval.

## Data Availability

The data presented in this study are available on request from the corresponding author, subject to institutional and ethical restrictions on patient data sharing. The complete analysis code, from the raw source extract to all reported estimates, is provided as Supplementary File S1.

## Supplementary Materials

The following supporting information can be downloaded at: https://www.mdpi.com/article/10.3390/jcm15218422/s1. File S1: Documented Python code reproducing the deduplication, data cleaning, primary and sensitivity analyses, paired DeLong comparisons, and patient-level bootstrap procedures from the raw source extract.

## Abbreviations

The following abbreviations are used in this manuscript:

ADQI	Acute Disease Quality Initiative
AIC	Akaike information criterion
AKI	Acute kidney injury
AKI-EPI	Acute Kidney Injury–Epidemiologic Prospective Investigation
APACHE II	Acute Physiology and Chronic Health Evaluation II
AUC	Area under the receiver operating characteristic curve
CAR	C-reactive protein-to-albumin ratio
CI	Confidence interval
CKD	Chronic kidney disease
COVID-19	Coronavirus disease 2019
CRP	C-reactive protein
eGFR	Estimated glomerular filtration rate
FROG-ICU	French and European Outcome reGistry in Intensive Care Units
ICD-10	International Classification of Diseases, Tenth Revision
ICU	Intensive care unit
IQR	Interquartile range
KDIGO	Kidney Disease: Improving Global Outcomes
MIMIC-III	Medical Information Mart for Intensive Care III
OR	Odds ratio
RECORD	REporting of studies Conducted using Observational Routinely-collected health Data
ROC	Receiver operating characteristic
SD	Standard deviation
SOFA	Sequential Organ Failure Assessment
STROBE	Strengthening the Reporting of Observational Studies in Epidemiology
